# Editorial: Soil-root-microbe interactions promote soil and plant health

**DOI:** 10.3389/fmicb.2023.1155234

**Published:** 2023-03-06

**Authors:** Bo Sun, Andrew D. Barnes

**Affiliations:** ^1^State Key Laboratory of Soil and Sustainable Agriculture, Institute of Soil Science, Chinese Academy of Sciences, Nanjing, China; ^2^Te Aka Mātuatua - School of Science, University of Waikato, Hamilton, New Zealand

**Keywords:** soil health, microbial interaction, microbial composition, microbial diversity, keystone taxa, rhizosphere, soil functions

Soil health is considered to be a central requirement of a sustainable, functioning agroecosystem (Al-Kaisi and Lowery, 2017). Since the 2010s, major programs to improve soil health have been launched in the USA (Honeycutt et al., 2020) and China (Sun et al., 2022). More recently, the European Commission set a mission in the 2021 Horizon Europe program to ensure the health of soil and provision of food. These high-level programs aim to address the current and future challenges of long-term food security and sustainable use of terrestrial ecosystems. Soil health is defined as the capacity of soil to function as a living system, within ecosystem and land-use boundaries, to sustain plant and animal productivity, maintain or enhance water and air quality, and promote plant and animal health (Doran and Zeiss, 2000). It is a holistic concept, owing to the many chemical, physical and biological properties of soil, as well as to the many processes occurring in soils and the functions they provide. Bottom-up and top-down forces can both influence soil health through mediating belowground biological functioning (Figure 1).

**Figure 1:**
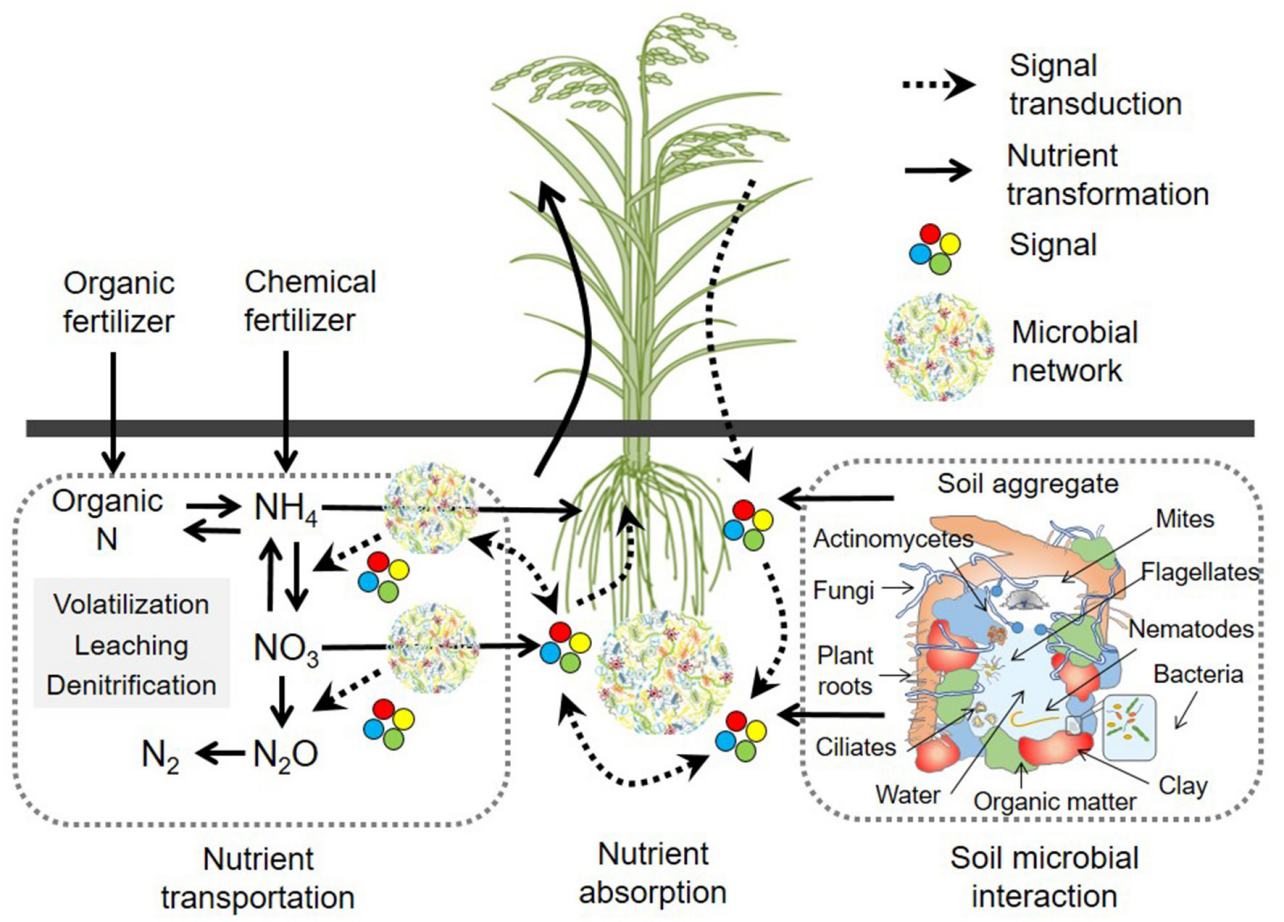
The impact of biotic interactions on soil nutrient transformation in agricultural systems.

Top-down forces imposed by soil predators can generate trophic cascades, which are caused by a change in some factor(s) affecting the survival or productivity of higher trophic levels of a food web and often manifest as inverse changes in abundance or biomass between adjacent trophic levels. Such trophic interactions can be targeted to modify specific functional soil biological groups to achieve the purpose of improving soil biological functioning (Jiang et al., 2017, 2020). However, experimental research and implementation of such manipulations are very scarce. Indeed, the lack of submissions to this Research Topic that investigate how top-down forces can influence soil health is testament to this scarcity.

In contrast, bottom-up cascades occur when a change in abiotic and biotic factors leads to changes in equilibrium abundances at the bottom of the food web, which cascades upward to all trophic levels. Soil pH is one of the most important environmental abiotic factors affecting soil biological metabolism, biodiversity and health (Luan et al., 2023). In acidic and alkaline soils, Fudjoe et al. and Zheng et al. found that organic material amendments optimized soil pH, respectively. These environmental changes modified the diversity and composition of functional microbial communities (ammonia oxidizers and denitrifiers) in the soil, and altered their potential interactions with keystone taxa which play an important role in determining network stability and modularity in co-occurrence networks. These dynamics of microbial interactions determine the rates of potential nitrification and denitrification in soil, which affected plant health and soil nitrogen supply. In addition to abiotic factors, environmental biotic factors—such as the diversity and identity of plant species—also determine the assembly of soil microbial communities and below-ground ecosystem services. Sun, Zhang, et al. revealed that rice crops under low phosphorus conditions actively enhanced rhizosphere bacterial and fungal phosphorus-mobilizing taxa to response to low phosphorus stress. In contrast, Sun, Chen, et al. found that mixed-cropping of different aromatic plants resulted in the soil chemical diversity that triggered significant turnover of the soil microbiome. This reshaped microbiota controls soil nutrient cycling and further benefits apple tree growth and fruit quality during the fruit development period. These studies give us significant, practical insight into how to maintain soil health in intensive farmland: utilization of plant-driven belowground microbiota for the continued capacity of soil functioning as a vital, living ecosystem that sustains plants in agroecosystems. Of course, the manipulation of soil biota through plants also requires the consideration of season-induced plant development. Results from Li et al. demonstrated that plant metabolism, which is affected by seasonal temperature and soil moisture, periodically enriches specific microbial taxa that are involved in carbohydrate degradation and nitrogen fixation. This rhythmic switching of plant rhizosphere carbon and nitrogen cycling functions is key to ensuring plant growth and nutrient uptake.

According to the five published research papers in the issue “*Soil-root-microbe interactions promote soil and plant health*,” we found most studies focused on the bottom-up control of inter-kingdom interactions for soil functioning and plant productivity by field management (such as addition of exogenous organic materials and plant diversification). In comparison, research on the contribution of top-down control to soil and plant health is relatively limited. Here, we emphasized the need to further develop a framework that integrates top-down and bottom-up processes in soil interaction networks (Barnes et al., 2020), capable of being used in different scenarios to understand the biological mechanisms that contribute to the construction of healthy soil for sustainable agriculture development.

## Author contributions

BS wrote the original manuscript. AB critically reviewed the manuscript. All authors contributed to the article and approved the publishment.
